# Virtual Issue on SARS-CoV-2: Evolution of a Pandemic

**DOI:** 10.1093/gbe/evad057

**Published:** 2023-04-19

**Authors:** Casey McGrath

In the last 3 years, the COVID-19 pandemic has touched every corner of the globe, resulting in substantial mortality, a number of chronic health problems, and long-lasting economic repercussions. The pandemic also had a profound effect on how scientific data are reported and disseminated, as researchers rushed to share their findings and discovered new ways to collaborate despite the challenges posed by lockdowns and other disruptions. Throughout this period, *Genome Biology and Evolution* welcomed papers that deepen our knowledge of SARS-CoV-2, the virus that causes COVID-19. The journal's Virtual Issue on SARS-CoV-2 collates some of the key papers published in *GBE* that have helped to shed light on the origins and evolution of this world-changing virus.

The first in the Virtual Issue is a recent study by Petrone et al. (2023) that analyzed differences in reporting delays among SARS-CoV-2 variants of concern (VOCs). The authors found that the reporting of genomic data for the Mu variant was significantly slower than that of other VOCs and that this trend could be observed across different countries, states, and even individual laboratories. Perhaps due in part to this reporting bias, Mu was not designated as a VOC until approximately 1 year after it emerged, which is substantially longer than the Delta and Omicron VOCs. According to Mary E. Petrone, a lead author on the study who has since completed her PhD from Yale University and is now at the University of Sydney, the researchers originally noticed this bias when reporting their own genomic data for SARS-CoV-2 to the Global Initiative on Sharing Avian Influenza Data (GISAID), which expanded their database to include SARS-CoV-2. “We noticed that a large number of our submissions to GISAID were getting rejected due to perceived quality control issues. When we took a closer look, we realized that a substantial portion of the rejected sequences were the Mu variant.” This may be because Mu carries a four-nucleotide deletion in ORF3a that causes a frameshift mutation that may often be flagged as a sequencing error. Fortunately, Mu did not spread globally to the same extent as Delta and Omicron. However, Petrone notes that such reporting biases must be addressed in order to avoid a delayed response to a VOC in the future: “We hope that our study will serve as a motivating factor to reduce genomic surveillance gaps for SARS-CoV-2 and other viruses of public health importance.”

Another recent article comes from Aiewsakun et al. (2023), who investigated the adaptation of SARS-CoV-2 to three cell lines that are commonly used to grow viral stocks for laboratory studies of the virus. They identified “numerous potential cell culture adaptation changes scattered across the entire virus genome.” In order to avoid confounding effects of these changes on lab experiments, the authors emphasize that it is important to perform deep-sequencing of cultured viral samples when performing sensitive SARS-CoV-2 experiments. They also noted that, among the three cell lines tested, Vero E6/TMPRSS2 cells induced the fewest changes and thus might be the best option for SARS-CoV-2 culture.

In addition to cell culture studies, animal models of SARS-CoV-2 infection and COVID-19 disease are critical for understanding how to prevent and treat the worst outcomes. Whereas the Syrian hamster is widely used as a model for mild-to-moderate COVID-19, the Roborovski dwarf hamster has become an important model for severe-to-lethal COVID-19. Although this model was previously limited by the absence of a full genome annotation, Andreotti et al. (2022) have provided a draft genome assembly that includes gene annotations and that “can now serve as a key resource for many research projects employing this hamster species.”

Two papers in the Virtual Issue investigate SARS-CoV-2 genotypes and polymorphisms in order to reveal patterns of viral diversity. Moustafa and Planet (2021) developed a tool called GNUVID (GNU-based Virus Identification) that integrates whole-genome multilocus sequence typing and machine learning and used it to assign all high-quality SARS-CoV-2 genomes available from GISAID to sequence type profiles. Their results revealed introduction and exportation events across US states and provided spatiotemporal estimates of effective viral diversity. This information could be used in the future to understand the effects of various interventions such as vaccines, travel restrictions, and mask mandates on the variation in circulating viruses. In their study, Zhu et al. (2021) investigated SARS-CoV-2 single-nucleotide polymorphisms (SNPs) that rapidly increased or decreased in frequency among clinical isolates in the initial 4 months of the pandemic. The SNPs that increased in frequency may have enhanced viral replication and disease severity, portending the appearance of new variants that later emerged.

Additional studies on SARS-CoV-2 mutation, recombination, and selection shed light on the virus's origins, as well as its future evolutionary trajectory. Lytras et al. (2022) performed a comprehensive phylogenetic study of SARS-CoV-2 and all related bat and pangolin sarbecoviruses sampled so far. Phylogenetic analyses revealed evidence of recombination among lineages, with a hotspot near the gene encoding the Spike protein. The results of this study further confirmed that horseshoe bats in the genus *Rhinolophus* are the likely reservoir species for the SARS-CoV-2 progenitor but that a direct proximal ancestor to SARS-CoV-2 had not yet been sampled. Consistent with these findings, Goldstein et al. (2022) found that SARS-CoV-2 and related coronaviruses commonly recombine with highly diverged viruses from unsampled or undersampled lineages, even in regions of the genome that are typically highly conserved. In several cases, it was clear that the viruses had recombined with strains that remain as-yet undiscovered and unrepresented in genetic databases, demonstrating how limited our current understanding and characterization of coronavirus diversity is.

Mutation is another mechanism by which SARS-CoV-2 generates diversity, allowing it to evolve and adapt. Morales et al. (2021) revealed that the true mutation rate of SARS-CoV-2 is likely to be approximately 50% higher than previous estimates. Earlier reports may have undercounted nonsense, missense, and synonymous mutations due to intrahost purifying selection. This has ramifications for estimating times to common ancestry and the potential for vaccine escape. De Maio et al. (2021) studied over 140,000 SARS-CoV-2 genomes and found that G-to-U and C-to-U substitutions accounted for the majority of mutations in the SARS-CoV-2 genome, which may reflect the activity of reactive oxygen species and APOBEC (Apolipoprotein B mRNA Editing Catalytic Polypeptide-like) proteins. APOBECs and ADARs (Adenosine Deaminases that Act on RNA) are host enzymes that edit the bases in double-stranded RNA. Indeed, a review article by Piontkivska et al. (2021) suggests that both APOBECs and ADARs act on SARS-CoV-2. Moreover, editing by these enzymes may be associated with enhanced production of inflammatory cytokines such as interferon, thus enhancing the virus's pathogenicity. Furthermore, according to a study by Ou et al. (2020), C-to-U substitutions may help to relieve the metabolic constraints on cytidine triphosphate, which is not only a building block in the SARS-CoV-2 genome but is also required for viral protein glycosylation, synthesis of the viral envelope, and the synthesis of host transfer RNAs. Targeting this key metabolic bottleneck may provide an avenue for future development of antiviral therapies.

The above studies have expanded our understanding of SARS-CoV-2 at a time when new data and insights were being published at an unprecedented pace. We hope that these papers will continue to inform and provide a foundation for future investigations into the evolution of the SARS-CoV-2 genome and viruses more broadly. Such analyses will bolster our ability to understand and predict epidemiological patterns, anticipate the emergence of new variants, develop effective countermeasures, and protect against new pandemic viruses.
